# The Potential of Consumer-Targeted Virtual Reality Relaxation Applications: Descriptive Usage, Uptake and Application Performance Statistics for a First-Generation Application

**DOI:** 10.3389/fpsyg.2019.00132

**Published:** 2019-02-04

**Authors:** Philip Lindner, Alexander Miloff, William Hamilton, Per Carlbring

**Affiliations:** ^1^Department of Psychology, Stockholm University, Stockholm, Sweden; ^2^Mimerse, Stockholm, Sweden; ^3^Department of Psychology, University of Southern Denmark, Odense, Denmark

**Keywords:** relaxation, virtual reality, stress, pain, consumer, application (app)

## Abstract

Virtual Reality (VR) technology can be used to create immersive environments that promote relaxation and distraction, yet it is only with the recent advent of consumer VR platforms that such applications have the potential for widespread dissemination, particularly in the form of consumer-targeted self-help applications available at regular digital marketplaces. If widely distributed and used as intended, such applications have the potential to make a much-needed impact on public mental health. In this study, we report real-world aggregated uptake, usage and application performance statistics from a first-generation consumer-targeted VR relaxation application which has been publicly available for almost 2 years. While a total of 40,000 unique users signals an impressive dissemination potential, average session duration was lower than expected, and the data suggests a low number of recurrent users. Usage of headphones and auxiliary input devices was relatively low, and some application performance issues were evident (e.g., lower than intended framerate and occurrence of overheating). These findings have important implications for the design of the future VR relaxation applications, revealing primarily that user engagement needs to be addressed in the early stage of development by including features that promote prolonged and recurrent use (e.g., gamification elements).

## Introduction

Virtual Reality (VR) refers to technology that simulates being present in a virtual, computer-generated world, most often achieved using a head-mounted display (HMD) that covers the user’s eyes with dual-display stereoscopy to simulate depth perception, withholding of the actual surroundings and making the video and audio presentation interactive to head movements (Botella et al., 2017; Freeman et al., 2017). Since the early 2000s (Hoffman et al., 2000), VR have been used to create immersive experiences for relaxation and distraction, with meta-analyses of clinical trials revealing that VR is effective in treating anxiety (Carl et al., 2018) and pain (Malloy and Milling, 2010; Kenney and Milling, 2016), and several studies have shown that VR can induce relaxation specifically (Riva et al., 2007; Baños et al., 2009, 2013, 2014; Valtchanov et al., 2010; Annerstedt et al., 2013; Serrano et al., 2016; Anderson et al., 2017).

To date, however, VR mental health interventions have seen very limited dissemination outside specialized clinics and university laboratories (Freeman et al., 2017). Arguably, the inaccessibility, high financial costs and low user-friendliness of the previous generation of VR hardware have constituted substantial barriers to dissemination (Segal et al., 2011; Schwartzman et al., 2012). Since 2016, consumer VR hardware and software has seen an impressive growth and now offers a mature ecosystem from development to distribution, in theory addressing past concerns and barriers (Lindner et al., 2018a). This makes consumer VR an attractive avenue to disseminate, on an unprecedented scale, immersive applications that reduce symptoms of stress, anxiety, and pain (Lindner et al., 2017), especially in the form of consumer-targeted applications running on consumer hardware and distributed through established digital marketplaces (Miloff et al., 2016; Donker et al., 2018; Freeman et al., 2018).

In the current study, we present real-world uptake data from a first-generation consumer-targeted VR relaxation and distraction application that has been publicly available on a digital marketplace for almost 2 years, providing a first glimpse of the dissemination potential of such applications, as well as valuable usage and application performance data that may guide the development of future applications.

## Materials and Methods

### Application

**FIGURE 1:**
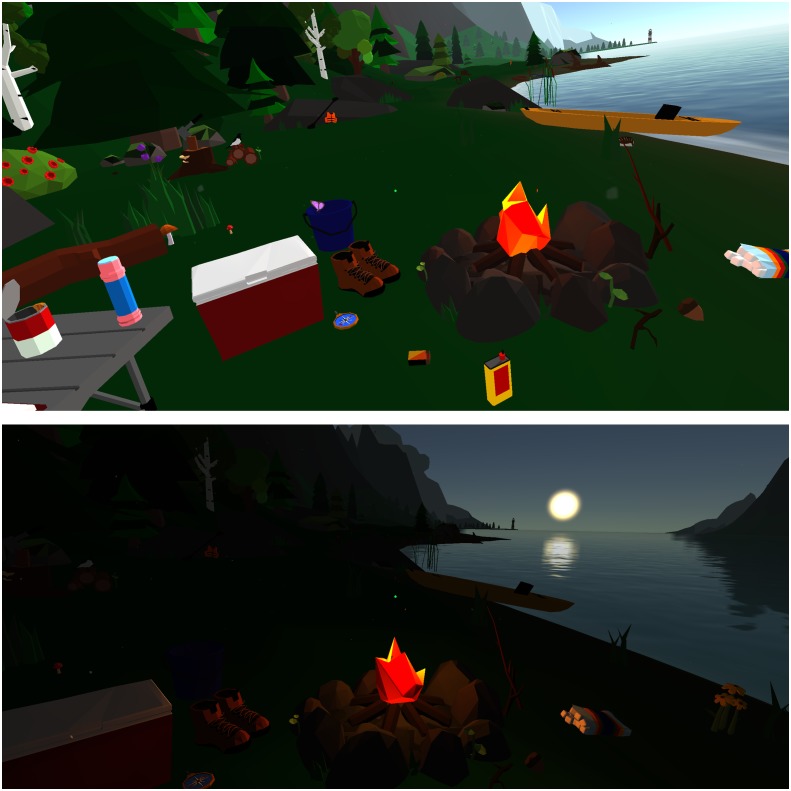
Screenshots of the Happy Place application. **(Top)** Day environment with interactive objects. **(Bottom)** Night environment. Screenshots published with permission from copyright holder Mimerse.

The Happy Place application was developed by Mimerse using the Unity game engine. Relaxation and distraction is induced by situating the user in a relaxing nature environment (Valtchanov et al., 2010; Annerstedt et al., 2013; Berto, 2014), one which includes full day-night and weather cycles, scripted animal behaviors, and spatialized background sounds. This virtual nature environment is stylized in a low-polygon appearance that is both visually pleasing and computationally less expensive to generate than photorealistic equivalents; an important concern for computationally constrained mobile VR units. See Figure 1 for screenshots. Using a gaze reticle interface, the user can explore the environment through head rotation: resting the reticle on one of 50 included objects will trigger minor environmental events. This as-requested interactivity aims to increase sense of presence and to provide users with the choice of a passive or active engagement style, making the application suitable for both relaxation and distraction. The user experience begins in an empty “void world” while the nature environment loads during approximately 30–50 s, which appears first through a saturated black and white filter before gradually coming to life and into full color during a 30-s transition, designed to rapidly increase immersion. Once the user has transitioned into the nature environment, the experience is open-ended and the user can remain as long as desired, with the option of listening to voice-over guided meditation.

### Data

The application was made publicly available for mobile VR users at no cost on the Oculus Store, in October 2016. Data from date of public release (1st week discarded to exclude live developer testing) were extracted from the developer platform. Raw data were aggregated on group level, meaning that there is no individual user data available for correlational or subgroup analyses. For presentation purposes, the data was then further aggregated (sum, mean, or max) on a weekly level to smooth out random and non-random (e.g., weekday) variations of no interest. Extracted metrics covered application uptake, usage and application performance: accumulated installations and uninstallations, unique active users and sessions (the later converted to a sessions per user ratio), average session duration, percentage usage of gamepad, controller and headphones, as well as average framerate (Frames Per Second, FPS; 60 intended), overheating rate (event percentage by session), available memory and battery burn rate. Oculus states that reported metrics have a maximum 5% error margin. Of note, some metrics were implemented during the data collection period.

By Swedish law (2003:460), ethical approval is not applicable to the handling or publishing of non-interventional human research data aggregated on group level (i.e., non-identifiable data). All users consented to having their uptake and basic usage data shared with Oculus, which in turn could be shared with third-parties including researchers, as part of the terms of service of the Oculus Store. Oculus granted permission to extract and publish the raw data, which is now available at an online repository (Lindner et al., 2018b). Thus, the current study describes analyses performed on publicly available data. While research on publicly available data (other common examples being, e.g., self-disclosed social media content, search statistics, etc.) is typically exempt from standard research regulations, ethical concerns have been raised (Metcalf and Crawford, 2016). These concerns do, however, not apply to aggregated data such as those presented in this report.

## Results

### Uptake Statistics

**FIGURE 2:**
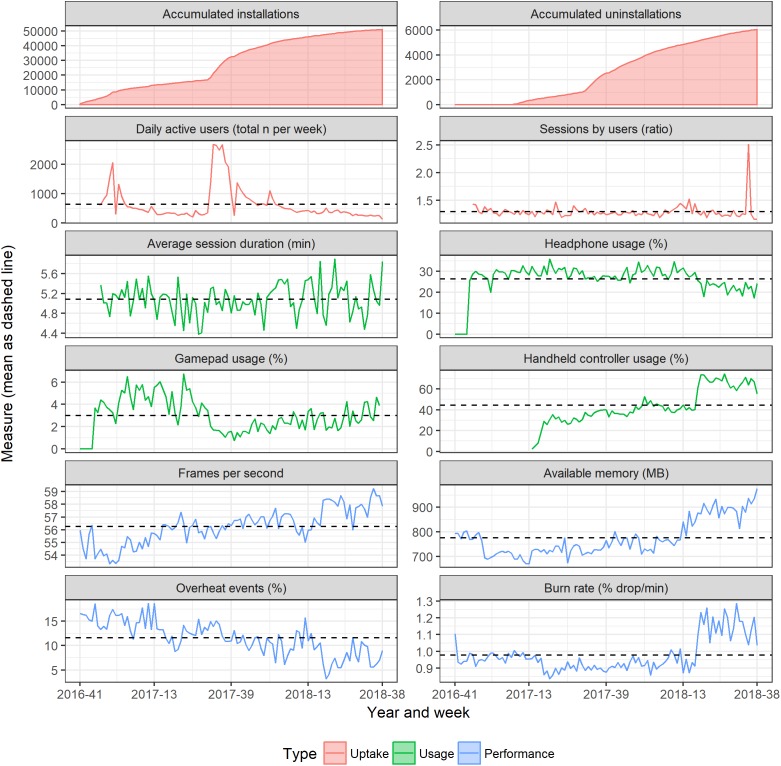
Uptake, usage and application performance statistics.

From October 2016 to September 2018, the application saw *n* = 40,153 unique active users, with daily active users (raw data) ranging from 4 to 653, and on an aggregated weekly level ranging from 123 to 2677 (which could be recurrent or new users). As evident by the increasing accumulated installations yet relatively stable number of daily active users (with the exception of two prominent spikes), there was a low degree of recurrent users. The ratio between number of sessions and active users was consistently around 1.3. The peaks in installations and active users is likely associated with increased media attention or in-store coverage, while the larger peak in session-per-user is likely random. See Figure 2 panel top rows.

### Usage Statistics

Average session duration was approximately 5 min. Headphone usage was stable at around 25%. Gamepad usage averaged around 3% during the period, with an apparent delayed decrease occurring after the introduction of handheld controllers in early 2017, the use of which increased over time. See Figure 2 panel middle rows.

### Application Performance Statistics

Average framerate increased over time and approached the intended 60 FPS in the final months. Available memory also increased, while overheating event rate decreased over time. Burn rate, however, increased during the last 6 months. See Figure 2 panel bottom rows.

## Discussion

From a dissemination perspective, the Happy Place application can be considered a successful first attempt at distributing consumer VR relaxation applications at an unprecedented scale. Over 40,000 unique users over a 2-year period is well beyond even the largest stress reduction trials, both VR-based (Serrano et al., 2016) and internet- or smartphone-based (Shimazu et al., 2005; Heber et al., 2016). These uptake number provides a glimpse of how many individuals can be reached with this type of intervention – a number which can be expected to rise with the continued growth of consumer VR.

The dissemination potential notwithstanding, application usage, indexed both by average session duration and daily users (which did not grow despite growing number of installations), was lower than expected. Without outcome data, individual data or known variances around the means, we can only speculate on whether the application was successful in inducing relaxation and pain distraction. Although the aim of the current study was not to examine efficacy or effectiveness, the low average session duration and low number of daily active user suggests that most users did not find the experience beneficial enough to use for prolonged durations or repeat regularly; however, this does not rule out the possibility of a subset of users having more frequent and longer session durations, indicative of effects. Further, our real-life data does not allow a differentiation between application engagement, acceptability and immersion, although they are likely highly inter-correlated; the measure of each of these aspects, and modeling of their moderating and mediating effects will need to be examined in a future study.

Usage issues can be addressed with application design: providing users with pre-set session duration options can help delivery of a minimum effective dose and will standardize usage, while repeated frequent use can be reinforced by outside-VR prompts (e.g., by email or a companion smartphone application), gamification elements in VR emphasizing progression (e.g., awarding points and badges for accomplishments), options to customize the virtual environment and session (sandbox, “More to explore,” design), and other features already ubiquitous in games and smartphone applications (Domínguez et al., 2013). Of importance, even with suboptimal adherence and retention, VR interventions that show small effect sizes at group level may achieve a large public health impact if distributed at scale. In addition, the risk of negative effects in VR are generally low (Fernández-Álvarez et al., 2018) and there is to our knowledge no suggestion in the extant literature that potential failed attempts at self-help translates into a reluctance to seek professional help.

Findings on the three metrics covering usage of input and output devices (gamepad and controller, and headphones, respectively) provide insights that should guide the development of future applications. The relatively low usage of headphones reveals that most users either rely on the built-in speaker or mute the audio. Thus, including high-quality, detailed audio, or making audio an integral but implicit part of the user experience is not advisable at present. Prompts may increase headphone use and future HMDs with built in high-quality headphones may alleviate the issue. As to the use of external input devices, use of both gamepads and controllers was low, which is not surprising given that the application was not designed for such input devices. Handheld controllers, the movements of which can be translated into virtual hands, have the potential to increase presence through increased interactivity. Since all modern VR platforms now feature handheld controllers, future research should explore how to best use this technology to increase relaxation and distraction effectiveness.

Finally, since the application was not continuously updated to increase application performance, the observed increases in FPS and available memory over time, and decrease of overheating events, likely reflect the release of more powerful devices during the data collection period. The slight increase in battery burn rate, which was moderate in relative terms but very small in absolute terms, likely mirrors the increased performance at expense of burn rate, as well as a changing device pool. More powerful devices will allow for more stable and content-rich applications, yet the very presence of overheating events and suboptimal framerates stress the importance of taking computational limitations of the intended device into account at an early stage in development.

## Strengths and Limitations

The primary limitation of the current study is that no individual data were available, meaning that between-subject variations in metrics could not be calculated and that associations between metrics cannot be examined. Also, only a limited number of metrics automatically collected by the Oculus platform, rather than the application itself, were available. Oculus estimates a 5% maximum error margin, an independent verification of which is not possible since no individual data is available. The analgesic efficacy of the application is currently being evaluated in a pilot randomized controlled trial (NCT03762213).

These limitations notwithstanding, this study reports unique, real-world uptake, usage and application performance data that could only have been estimated using other methods, from a large number of users (forty thousand), and over a relatively long duration (almost 2 years).

## Conclusion

We conclude that consumer VR relaxation applications do indeed present an attractive opportunity for unprecedented dissemination that can achieve a much-needed impact on mental public health problems such as stress, anxiety and pain. However, application design must also be guided by both psychological science and real-world usage data, and there is a constant need for research on efficacy, effectiveness and mechanisms of action.

## Author Contributions

PL extracted and analyzed data, and drafted the manuscript. WH developed the application. AM, WH, and PC made significant contributions to the interpretation of findings and writing.

## Conflict of Interest Statement

PL consults for Mimerse, the application developer, but holds no financial stake in the company (a private limited company). WH is the founder, owner, and Chief Technology Officer of Mimerse. The Happy Place application described in this report does not generate revenue for Mimerse since it was released free of charge. The remaining authors declare that the research was conducted in the absence of any commercial or financial relationships that could be construed as a potential conflict of interest. The handling Editor declared a past co-authorship with one of the authors PC.
